# Host Genus and Habitat Use Shape the Distribution of *Batrachochytrium dendrobatidis* Lineages in a Hyper‐Diverse Tropical Amphibian Community

**DOI:** 10.1002/ece3.73250

**Published:** 2026-03-22

**Authors:** Shannon Buttimer, Wesley J. Neely, Jack M. Boyette, Carolina Lambertini, Renato Martins, Karen A. Paniagua Torres, David Rodriguez, C. Guilherme Becker

**Affiliations:** ^1^ Department of Biology The Pennsylvania State University University Park Pennsylvania USA; ^2^ One Health Microbiome Center, Center for Infectious Disease Dynamics, Huck Institutes of the Life Sciences The Pennsylvania State University Pennsylvania USA; ^3^ Department of Biology Texas State University San Marcos Texas USA; ^4^ Department of Biodiversity, Aquaculture Center (CAUNESP), and CBioClima, I.B Universidade Estadual Paulista Rio Claro São Paulo Brazil

**Keywords:** amphibian, *Batrachochytrium dendrobatidis*, chytridiomycosis, genotype, tropical ecology

## Abstract

Pathogens often exploit ecological and evolutionary opportunities created by anthropogenic change, with profound consequences for host communities. In Brazil's Atlantic Forest, the amphibian chytrid fungus *Batrachochytrium dendrobatidis* (Bd) exemplifies this dynamic, with two co‐occurring lineages: the enzootic Bd‐Brazil lineage and the invasive Global Panzootic Lineage (Bd‐GPL), implicated in historical amphibian declines and Bd hybridization events. To investigate how host taxonomy and habitat use influence Bd lineage distribution, we sampled 3836 amphibians representing 42 species across paired aquatic and terrestrial transects over a two‐year period. We successfully genotyped *n* = 252 out of 777 Bd‐positive samples using nuclear and mitochondrial SNP assays to differentiate between Bd‐GPL, Bd‐Brazil, hybrids, and coinfections. Our results reveal that Bd lineage distribution is nonrandomly associated with host genus and habitat type. Stream‐dwelling frogs, particularly those in the genus *Hylodes*, had higher rates of coinfection with Bd‐GPL and Bd‐Brazil than most other genera. This pattern may reflect their lifelong association with streams, which might increase their exposure to zoospores from multiple Bd lineages. In contrast, terrestrial transects were dominated by single‐lineage Bd‐GPL infections, even when accounting for differences in amphibian species composition and host genus among transect types. These findings suggest that aquatic habitats could serve as refugia for Bd‐Brazil, while Bd‐GPL may exhibit more desiccation tolerance. Methodological limitations of this study, including biases towards successfully genotyping high‐load infections and limited genomic resolution, underscore the need for more high‐resolution sequencing approaches to fully understand pathogen dynamics in the Atlantic Forest.

## Graphical abstract

Anthropogenic change has facilitated the spread of the amphibian chytrid fungus *Batrachochytrium dendrobatidis* (Bd) in Brazil's Atlantic Forest, where two Bd lineages (Bd‐GPL and Bd‐Brazil) co‐occur. We successfully genotyped 252 Bd‐positive samples from a diverse amphibian assemblage spanning aquatic and terrestrial habitats and found that Bd lineage distribution varies by host genus and habitat type, with coinfections most common in stream‐dwelling frogs like *Hylodes*. These results suggest that habitat and host heterogeneity may support the persistence of certain pathogen lineages.

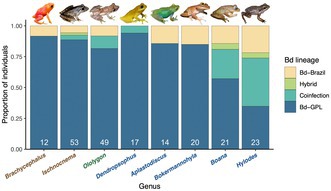

## 1 Introduction

Globalization is increasingly recognized for its role in promoting emerging infectious diseases (EIDs) in wildlife (Cunningham et al. 2017; Daszak et al. 2001; Tazerji et al. 2022). Specifically, the anthropogenic translocation of pathogens drives both the introduction of novel infectious agents to previously unaffected regions and the spread of new genetic variants of existing pathogens (Marie and Gordon 2023; O'Hanlon et al. 2018). This process, driven by human activities such as trade, travel, and habitat modification, can lead to significant ecological and epidemiological consequences, including the global dispersal of pathogens, regional disease outbreaks, and altered host‐pathogen dynamics (Aguirre 2017). The amphibian fungal pathogen *Batrachochytrium dendrobatidis* (Bd) illustrates how globalization has enabled the panzootic spread of a wildlife disease, with heterogeneous outcomes in different areas of the world, especially in biodiverse ecosystems (Fisher and Garner 2020; Jenkinson et al. 2016; O'Hanlon et al. 2018; Rosenblum et al. 2013). These divergent outcomes reflect intricate interactions between Bd genotypes, host diversity, and local environmental conditions that collectively determine disease severity in amphibian communities.

Bd most likely originated in East Asia, as evidenced by a lack of recent regional Bd‐related amphibian declines, a high tolerance exhibited by native hosts, and the identification of the ancestral Bd‐Asia‐1 lineage (Bai et al. 2012; Bataille et al. 2013; Fu and Waldman 2019; O'Hanlon et al. 2018; Sun et al. 2025). Over evolutionary time, Bd diversified into multiple phylogenetically distinct lineages, with at least five major clades identified to date (Farrer et al. 2011; Rosenblum et al. 2013). The Global Panzootic Lineage (Bd‐GPL) emerged more recently and has been implicated in most contemporary amphibian declines on all continents where amphibians occur except Asia (Farrer et al. 2011; James et al. 2009; O'Hanlon et al. 2018; Scheele et al. 2019). Other lineages, such as Bd‐Asia‐2/Brazil (hereafter Bd‐Brazil), are highly divergent, have more restricted distributions, and are often considered “enzootic” to their regions (Jenkinson et al. 2016; Rosenblum et al. 2013; Schloegel et al. 2012). The spread of these lineages has been facilitated by human activities, namely amphibian trade (Schloegel et al. 2012). Abiotic factors such as temperature affect Bd's growth rate, zoospore production, and infectivity, thus playing a key role in determining lineage fitness and potentially distribution (Sheets et al. 2021; Sun et al. 2025; Voyles et al. 2017). Current distributions of Bd lineages likely reflect a combination of invasion history, their genetic capacity to adapt to local environmental conditions, and their ability to overcome endemic host defenses (Belasen et al. 2022).

Brazil represents a uniquely complex system for studying Bd biology and evolution. While other countries in South America have been shown to only harbor the invasive Bd‐GPL lineage (Byrne et al. 2019; James et al. 2015; Neely et al. 2025; Smart et al. 2024), both Bd‐GPL and Bd‐Brazil are present in Brazil's Atlantic Forest (Byrne et al. 2019; O'Hanlon et al. 2018). In this region, Bd‐GPL is widespread and geographically unstructured, suggesting the lineage underwent recent introduction and rapid spread (Jenkinson et al. 2016). In contrast, Bd‐Brazil demonstrates strong geographic and genetic structure, indicating a relatively long history of regional endemism (over 100 years; Jenkinson et al. 2016; Rodriguez et al. 2014). Such historical endemism may have allowed for pathogen attenuation to occur through co‐evolution with Brazilian amphibians or, alternatively, mild virulence may reflect the ancestral state of Bd‐Brazil (Belasen et al. 2022; Jenkinson et al. 2016; Rodriguez et al. 2014). This mild virulence phenotype is evidenced by two independent laboratory studies focusing on the Brazilian frog genus *Brachycephalus*, in which infections with Bd‐GPL were characterized by higher Bd loads and a greater likelihood of host mortality than infections with Bd‐Brazil (Greenspan et al. 2018; McDonald et al. 2023). Given that Bd‐Brazil appears to have historically been present in the Atlantic Forest and is generally associated with lower mortality rates in native amphibians, most evidence points to Bd‐GPL having caused the amphibian die‐offs documented in the 1970s and 1980s (Carvalho et al. 2017; Eterovick et al. 2005; Heyer et al. 1988; Rosenblum et al. 2013; Toledo et al. 2023; Weygoldt 1989).

Pathogen dynamics in Brazil were further complicated by the unexpected discovery of recombinant hybrid Bd lineages in the wild, as Bd was previously thought to be strictly asexual (Jenkinson et al. 2016; Nieuwenhuis and James 2016; Rosenblum et al. 2013; Schloegel et al. 2012). Hybrids of Bd‐GPL and Bd‐Brazil demonstrate higher virulence than either parental lineage when tested on native hosts such as *Brachycephalus rotenbergae* (Greenspan et al. 2018). Interestingly, while Bd‐GPL tends to outcompete Bd‐Brazil in coinfections, Bd‐Brazil persists in much of the Atlantic Forest (Carvalho et al. 2023; Jenkinson et al. 2018). This raises questions about how Bd‐Brazil continues to persist despite its apparent competitive disadvantage against Bd‐GPL.

Recent work by Byrne et al. (2022) suggests that certain Bd sub‐lineages may preferentially infect and/or have co‐evolved with certain North American host species, including *Rana catesbeiana* , which was introduced to both the western United States and Brazil (Lever 2003; Schloegel et al. 2012). Host specificity of Bd genotypes may have significant implications for Bd dynamics in the amphibian species‐rich Brazilian Atlantic Forest, where lineages may segregate among host species, minimizing competition and enabling persistence of less competitive lineages such as Bd‐Brazil. The local amphibian community includes a mix of terrestrial direct‐developing and aquatic larval‐developing species that occupy a wide range of habitats, such as streams, ponds, trees, and the forest floor (Haddad et al. 2013). This variability provides an opportunity to test for associations between the presence of certain Bd lineages and host species, developmental mode, and preferred habitat type.

Despite extensive research, the ecological and evolutionary factors shaping the distribution and persistence of Bd‐GPL, Bd‐Brazil, and their hybrids within this diverse amphibian community remain poorly understood. Here, we predicted that Bd lineages would exhibit nonrandom associations with certain host genera, developmental modes, and habitat types. To investigate this, we sampled an amphibian community in the state of São Paulo, Brazil, bimonthly over a two‐year period. We collected over 3000 skin swabs to sample and quantify Bd along fourteen paired transects in both aquatic and terrestrial habitats and quantified Bd loads of skin swabs. For the subset of samples that tested positive for Bd, we used nuclear and mitochondrial single‐nucleotide polymorphism (SNP)‐based assays, which, when combined, can differentiate between Bd‐GPL, Bd‐Brazil, hybrids, and coinfections (Carvalho et al. 2023; Jenkinson et al. 2018). Our study provides new insights into host specificity and Bd genotypic turnover in a highly diverse tropical amphibian community, with important implications for understanding pathogen persistence, hybridization, and amphibian conservation in the Atlantic Forest.

## 2 Materials and Methods

### 2.1 Study Site and Focal Amphibian Species

All samples were collected within the continuous Atlantic Forest in Parque Estadual Serra do Mar—Núcleo Santa Virgínia in São Paulo, Brazil, from December 2020 to January 2023. This site is composed of primary and secondary tropical coastal montane forest. The park contains at least 44 frog species (Gilbert et al. 2025; Haddad et al. 2013). Amphibians in the Atlantic Forest exhibit two main developmental modes: terrestrial direct development and aquatic larval development. Brachycephaloidea is a direct‐developing clade of over 1000 New World frogs well‐represented in the Atlantic Forest (Hedges et al. 2008; Padial et al. 2014). This group of frogs progress through the tadpole stage within eggs laid in the terrestrial environment and hatch as small froglets. Most other genera in the area are larval developers that lay eggs in aquatic environments (i.e., ponds or streams). A few indirect‐developing taxa, such as many *Ololygon* and *Dendrophryniscus* species, breed and lay their eggs in bromeliads, where they develop as tadpoles.

### 2.2 Fieldwork

**FIGURE 1:**
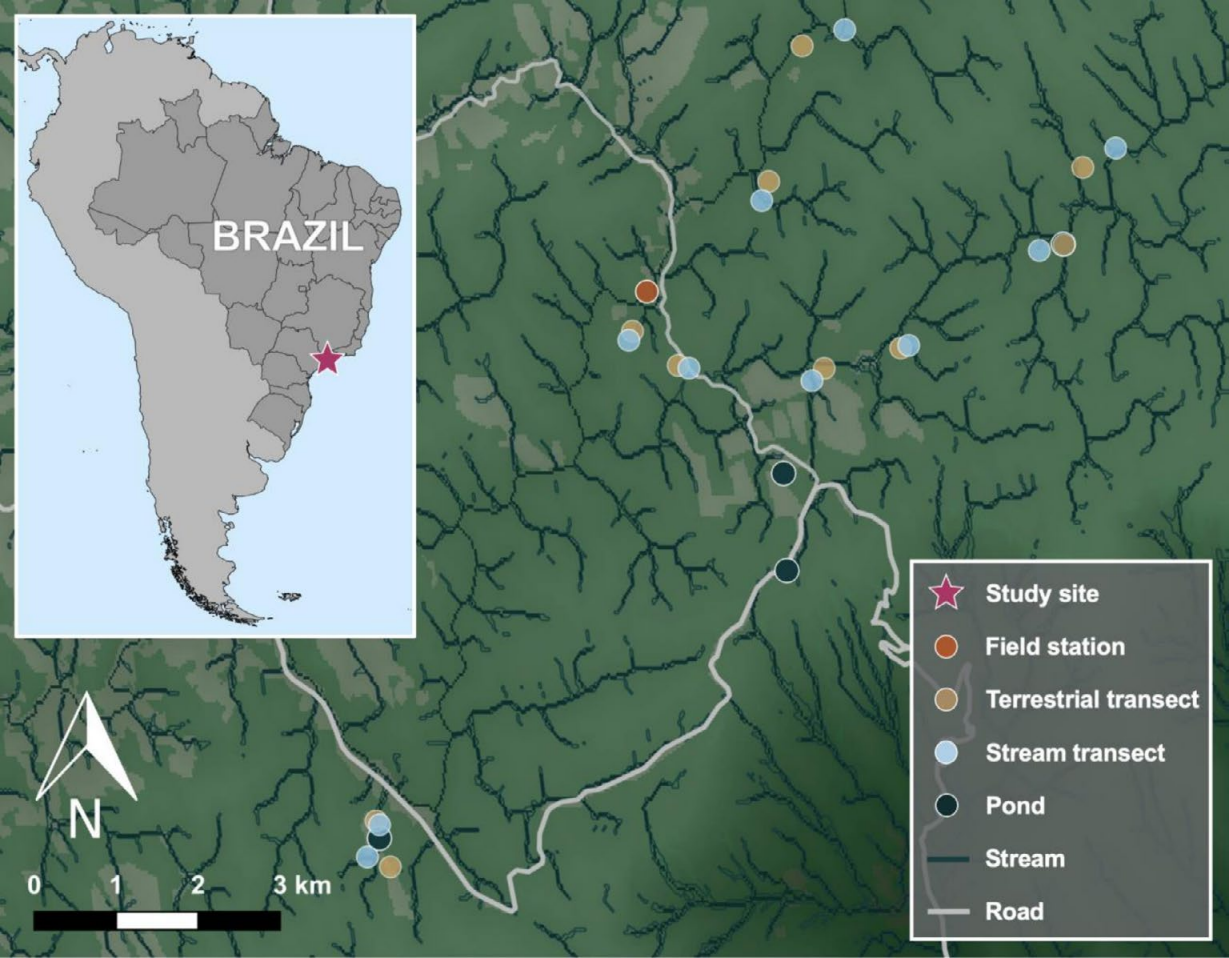
Map showing the locations of study transects in Parque Estadual Serra do Mar—Núcleo Santa Virgínia in São Paulo (SP), Brazil at local and continental scales. Transect points correspond to the end points of the 100‐m transects.

We established ten pairs of 100‐m‐long transects within a single fifteen‐km diameter area to minimize the effects of altitude on *Batrachochytrium dendrobatidis* infection dynamics (Becker and Zamudio 2011). Each pair included one stream transect and one terrestrial transect. Stream transects were comprised of one meter along each stream bank, and terrestrial transects were two meters wide and placed between twenty and fifty meters from the water, running parallel to the stream transects (Figure 1). Additionally, we sampled amphibians near three different ponds using the same methods as for stream transects. Distinct pairs of transects were separated by an average of one km and were between 901 and 1012 m above sea level. Since some frogs are diurnal and others are nocturnal, we conducted surveys during the day and at night to ensure a robust representation of the amphibian community. We conducted bimonthly campaigns consisting of three consecutive days and nights per transect pair. Due to logistical constraints, some transect pairs could only be sampled once per campaign.

While running transects, we listened for vocalizations and scanned for movement and eye shine to locate amphibians on the ground, in vegetation, and within streams or ponds. Upon capture with nitrile gloves or a clean plastic bag, we rinsed amphibians with distilled water (Culp et al. 2007) and then swabbed them with a sterile rayon swab (MWE 113) using standard protocols (Hyatt et al. 2007). We stored swabs on ice in sterile 1.5 mL tubes in the field and then transported them to a freezer for storage at −20°C. We captured a maximum of ten of each species at each transect during each sampling period. In total, we encountered 3836 amphibians, representing 42 different anuran species. All animal sampling was approved by IACUC (#202102112) and SisGen permits (#AC2F7B2 and #R42A06F). Sample sizes per campaign are shown in Table A1.

### 2.3 Sample Processing

#### 2.3.1 DNA Extraction

We extracted DNA from swabs using GMax Mini Genomic DNA Kits (IBI Scientific) with a slight alteration to the kit's protocol. We incubated the swabs with Proteinase K overnight at 60°C to increase DNA yield (Caligiuri et al. 2019). We randomly selected samples to extract each day to minimize biases based on sample collection date, and on each extraction date, we included a blank extraction control.

#### 2.3.2 *Batrachochytrium dendrobatidis* Quantification

To quantify Bd from aliquots of extracted DNA, we used a qPCR assay with Bd‐specific primers (Boyle et al. 2004) and synthetic Bd standards (Pisces Molecular) diluted from 10^6^ to 10^0^ ITS (Internal Transcribed Spacer 1) gene copies. We also used TaqMan Exogenous Internal Positive Control reagents (IPCs) to detect PCR inhibition and decrease the incidence of false negatives. Each reaction was composed of 12.5 μL Sensi‐Fast Lo‐ROX master mix (Meridian Bioscience), 1.13 μL of 20 μM ITS1‐3 primer (CCTTGATATAATACAGTGTGCCATATGTC), 1.13 μL of 20 μM 5.8S Chytr primer (AGCCAAGAGATCCGTTGTCAAA), 1.2 μL of 5 μM Chytr MGB2 probe (FAM labeled), 1 μL of 100× BSA, 0.83 μL of 10× Exo‐IPC mix (VIC labeled), 0.17 μL of Exo‐IPC DNA, 2.04 μL of nuclease‐free water, and 5 μL of template DNA or Bd standard, for a total volume of 25 μL per well. We ran qPCRs in singlicate on a QuantStudio 3 with the following program: 2 min at 50°C, 10 min at 95°C, then 50 cycles of 15 s at 95°C and 1 min at 60°C. We included a negative qPCR control well in each plate, none of which amplified. If the IPC from a well did not amplify, we repeated the qPCR for that sample. To calculate whole‐swab Bd loads, we multiplied loads by 20, then added 1 and log_10_‐transformed the counts to correct for non‐normal residual distributions in downstream analyses. Detailed analyses of Bd prevalence and loads by reproductive mode, habitat type, and precipitation can be found in Gilbert et al. (2025).

#### 2.3.3 *Batrachochytrium dendrobatidis* Genotyping

We attempted to genotype a total of 777 Bd‐positive amphibian skin swab samples from 19 different frog genera and 36 species. Some of our 781 Bd‐positive samples had too little remaining DNA for genotyping and thus were not included. To differentiate between Bd‐Brazil, Bd‐GPL, coinfections, and hybrid lineages, we used a combination of two qPCR‐based SNP assays. The first assay (BdSC9_621917) targets a nuclear marker and was developed and used by Carvalho et al. (2023). The second assay (Bdmt_26360) was developed by Jenkinson et al. (2018) and targets a mitochondrial marker. This allows for the identification of coinfections, since any single Bd genotype should only possess one mitochondrial genotype. Both assays used the same recipe: 5.0 μL of TaqMan Fast Advanced Master Mix (ThermoFisher), 0.5 μL of 20X assay (4 μM of each probe and 18 μM of each primer), 2.0 μL nuclease‐free water, and 2.5 μL of template DNA, for a total of 10 μL per well. Samples were run in 384‐well plates on a QuantStudio 5 using the following cycling program: 60°C for 30 s, 95°C for 20 s, then 50 cycles of 95°C for 1 s and 60°C for 20 s, followed by 60°C for 30 s. Every plate included at least one well of each representative Bd lineage in the study: Bd‐GPL (NAF‐01), a hybrid strain (CLFT‐024‐2), and Bd‐Brazil (BAF‐038). Each plate also contained a field control swab and a qPCR control, none of which amplified.

**FIGURE 2:**
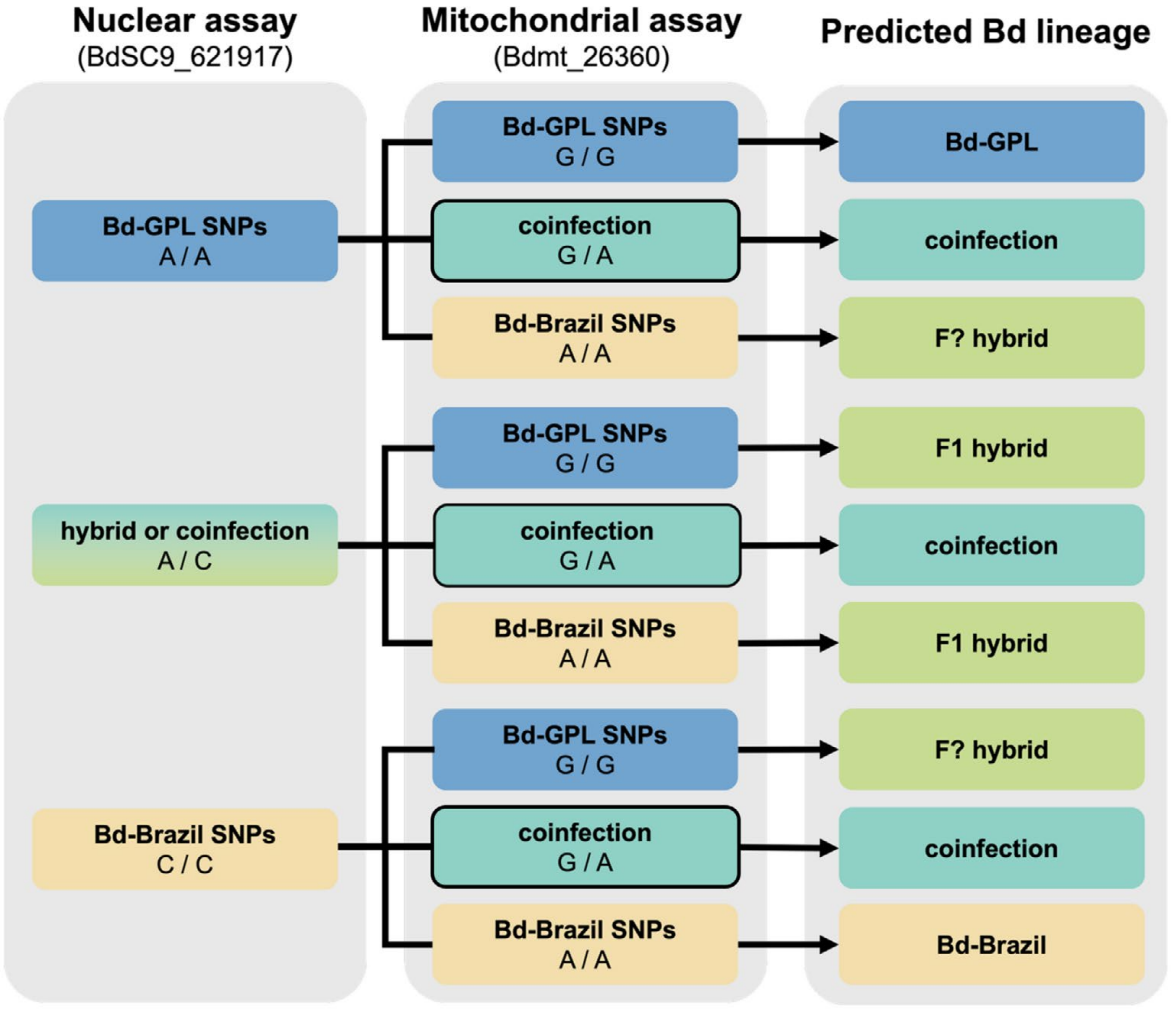
Predicted *Batrachochytrium dendrobatidis* genotypes based on the combined results from each SNP assay. Boxes are colored based on the lineage associated with each combination of alleles (blue: Bd‐GPL, teal: Coinfection, green: Hybrid, yellow: Bd‐Brazil). A black outline indicates that a call can be made using only the mitochondrial assay result.

We inferred Bd lineage by analyzing the combinations of nuclear and mitochondrial SNPs in each sample. In the nuclear assay, allele A is indicative of Bd‐GPL, whereas allele C is indicative of Bd‐Brazil. For the mitochondrial assay, allele G is indicative of Bd‐GPL and allele A is indicative of Bd‐Brazil. Because mitochondria are uniparentally inherited in Bd (Ghosh et al. 2021; Jenkinson et al. 2018), coinfections can be distinguished from hybrids if more than one mitochondrial SNP is detected. For this reason, samples that indicate the presence of both allele G and allele A can be determined to be coinfections even in the absence of a nuclear SNP call (Figure 2).

### 2.4 Statistical Analysis

We conducted all statistical analyses in R version 4.3.0 (R Development Core Team 2023). To visualize differences in amphibian species composition between transects, we estimated local frog species composition using capture rates. We filtered encounter data to include only transect pairs that were sampled three times per campaign to control for sampling effort. Then, we grouped the data by transect pair ID, transect type (i.e., terrestrial or aquatic), and sampling campaign and calculated the presence‐absence‐based Jaccard distance metric using the vegdist function from the *vegan* package (Oksanen et al. 2020). We then compared differences in species composition by transect pair ID and transect type using permutational multivariate analysis of variance (PERMANOVA) of the dissimilarity matrices using the adonis2 function and compared group dispersions with the betadisper function. Next, we performed principal coordinate analysis (PCoA) using the *ape* package (Paradis et al. 2024) and plotted differences in community composition using *ggplot2* (Wickham et al. 2022).

To determine the minimum Bd load at which amplification efficiency exceeded 75% (i.e., the load at which genotyping is likely to succeed), we used logistic regression to model amplification success as a function of Bd load. We classified amplification outcomes as binary (success = 1, failure = 0) for both nuclear and mitochondrial assays. We then fit separate logistic regression models for each assay using the glm() function with a binomial link. We identified the Bd load threshold corresponding to a predicted amplification probability of 75% using the uniroot() function.

To compare differences in the proportion of Bd genotypes between amphibian genera with more than 10 successfully genotyped swabs (i.e., *Brachycephalus, Ischnocnema, Dendrophryniscus, Ololygon, Dendropsophus, Aplastodiscus, Bokermannohyla, Boana, and Hylodes*), we employed pairwise chi‐squared tests for each genus pair.

Finally, to examine the relationship between Bd lineage, transect type, and developmental mode while controlling for differences in amphibian species composition at each transect/campaign (captured by Jaccard PCoA Axis 1), we ran binomial generalized linear mixed models (GLMMs) using the package *glmmTMB* (Brooks et al. 2017). The model formula was as follows: is_GPL ~ PCoA1_Jac + transect_type + developmental_mode + (1|transect_id) + (1|genus), where the response variable is_GPL is a binary indicator of whether a sample represented single‐lineage Bd‐GPL infection. We ran two additional models with is_Brazil and is_Coinfection as the response variables. We simulated model residuals and checked assumptions using the *DHARMa* package (Hartig and Lohse 2022).

## 3 Results

### 3.1 Amphibian Community Composition

**FIGURE 3:**
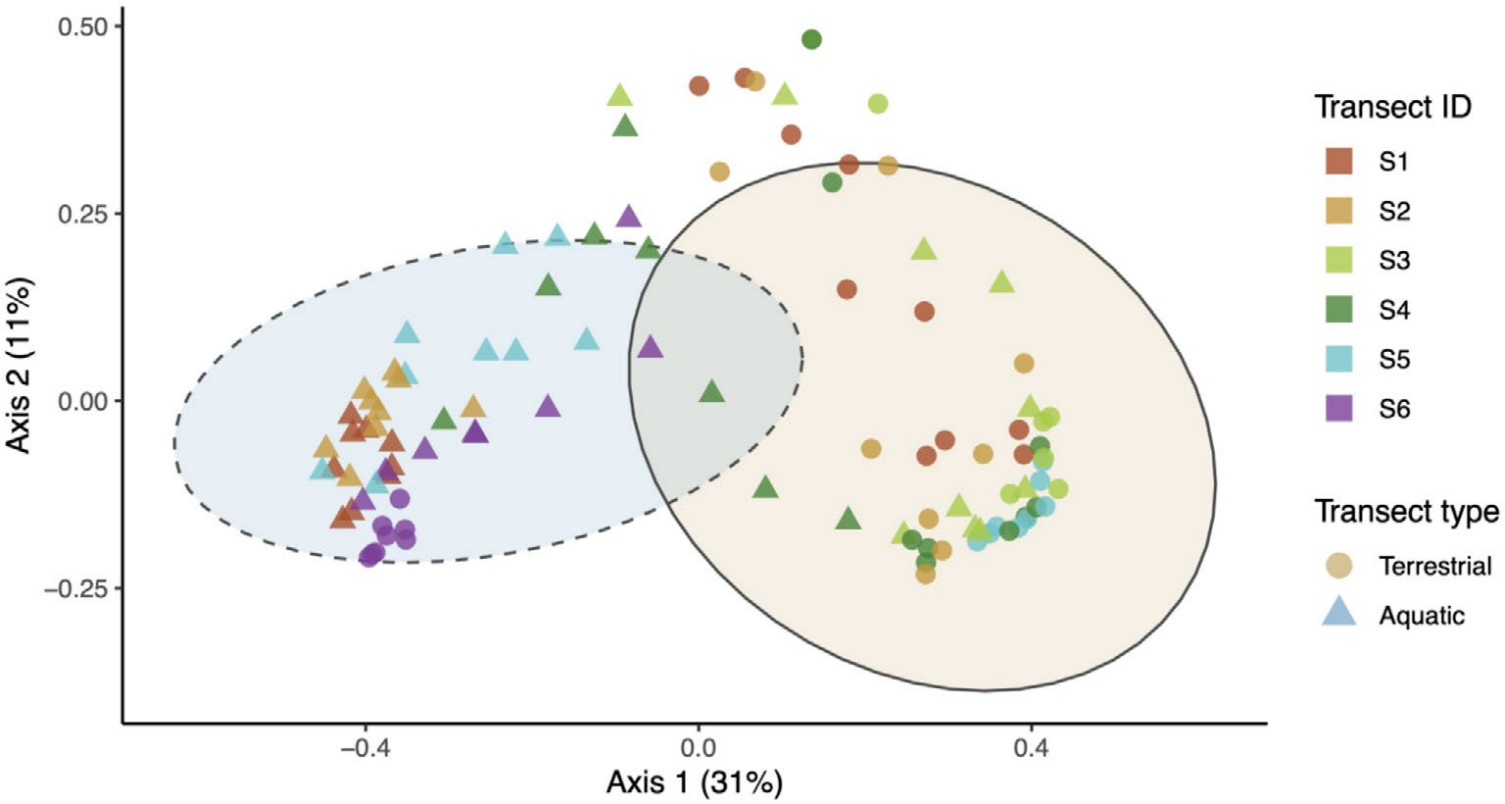
Principal coordinates analysis of amphibian species composition Parque Estadual Serra do Mar—Núcleo Santa Virgínia in São Paulo, Brazil using Jaccard distances with 80% confidence ellipses. Only transects with even sampling efforts are shown.

Frog species composition varied by transect type (*p* < 0.001), transect pair ID (*p* < 0.001), and the interaction between transect type and transect pair ID (*p* < 0.001; Figure 3; Table A2). Dispersion of frog species composition differed between transect pairs (Pseudo‐F = 5.476, *R* ^2^ = 0.218, *p* < 0.001) and between transect types, with lower variability in species composition along aquatic transects (Pseudo‐F = 28.072, *R* ^2^ = 0.216, *p* < 0.001).

We quantified the Bd loads of a total of 3404 samples using qPCR (Table A3). Bd loads ranged from zero to 4.06 **×** 10^7^ ITS gene copies. Overall Bd prevalence was 23% and average infection intensity was 3.19 **×** 10^5^ ITS copies.

### 3.2 *Batrachochytrium dendrobatidis* Genotyping

**FIGURE 4:**
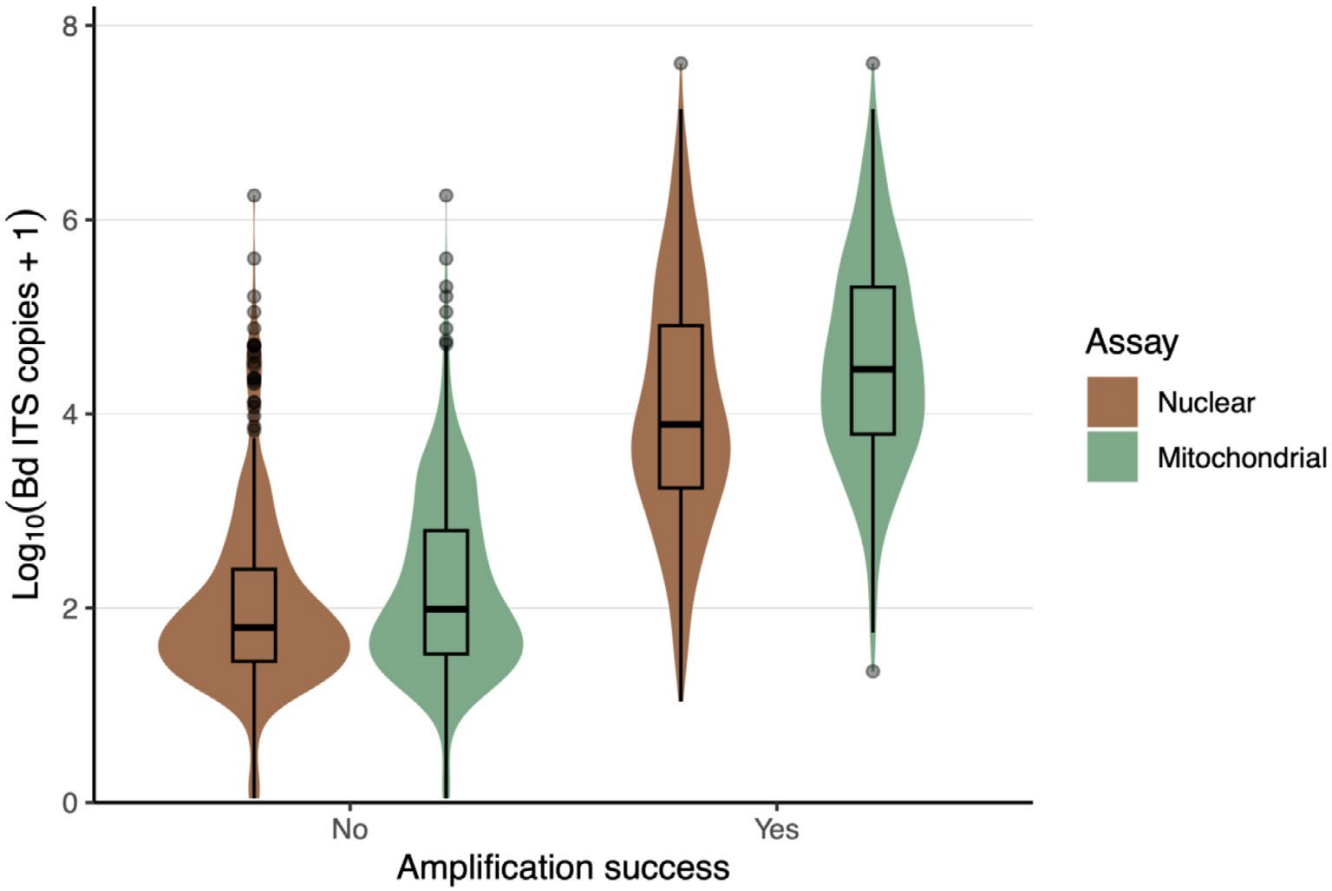
Violin plots showing the distribution of *Batrachochytrium dendrobatidis* (Bd) loads between samples that were unsuccessfully and successfully amplified using the nuclear and mitochondrial SNP assays. Samples with higher Bd loads were more likely to yield successful amplifications for both assays (*p* < 0.001).

**FIGURE A1:**
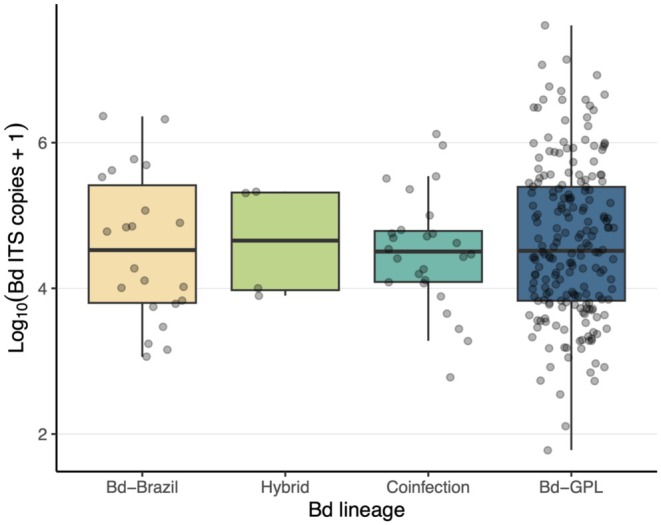
Boxplots showing the number of *Batrachochytrium dendrobatidis* (Bd) ITS gene copies detected on successfully genotyped swabs. Please note that there may be substantial ITS copy number variation between Bd lineages (Longo et al. 2013; Rebollar et al. 2017).

The *Batrachochytrium dendrobatidis* (Bd) loads of the swab samples used for genotyping ranged from 1 to 8.03 **×** 10^7^ ITS copies. We successfully genotyped 274 samples using the nuclear assay (efficiency = 35.2%) and 382 samples using the mitochondrial assay (efficiency = 49.1%). 153 samples had ambiguous calls and were excluded from downstream analyses. The Bd lineages present in 252 samples could be unambiguously called (overall success rate = 32.4%). Amplification success for both the nuclear (Student's *t*‐test: *t* = −31.208, df = 775, *p* < 0.001) and mitochondrial (*t* = −25.983, df = 775, *p* < 0.001) assays was associated with higher Bd loads (Figure 4). The minimum Bd load at which amplification efficiency exceeded 75% was approximately 1.34 **×** 10^4^ ITS copies for the nuclear assay and 3.86 **×** 10^3^ ITS copies for the mitochondrial assay. See Figure A1 for Bd loads (ITS copy number) by lineage.

**FIGURE 5:**
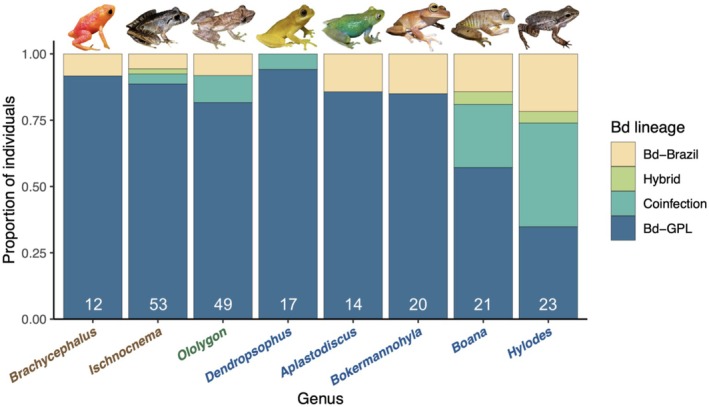
Stacked bar plot showing the proportion of swabs assigned to each *Batrachochytrium dendrobatidis* (Bd) lineage from genera of frogs with more than *n* = 10 successfully genotyped swabs. Names of genera are colored by reproductive mode (brown: Terrestrial direct development, green: Larval development in vegetation, blue: Larval development in aquatic habitats). The number of samples from each genus is shown in white. Significant (*p* < 0.05) pairwise chi‐squared comparisons of Bd lineage counts: *Aplastodiscus* versus *Hylodes* (*χ* ^2^ = 10.519, *p* = 0.015), *Boana* versus *Ischnocnema* (*χ* ^2^ = 10.099, *p* = 0.018), *Bokermannohyla* versus *Hylodes* (*χ* ^2^ = 13.597, *p* = 0.004), *Brachycephalus* versus *Hylodes* (*χ* ^2^ = 10.745, *p* = 0.013), *Dendropsophus* versus *Hylodes* (*χ* ^2^ = 14.493, *p* = 0.002), *Ischnocnema* versus *Hylodes* (*χ* ^2^ = 24.600, *p* < 0.001), *Hylodes* versus *Ololygon* (*χ* ^2^ = 16.328, *p* < 0.001).

**FIGURE A2:**
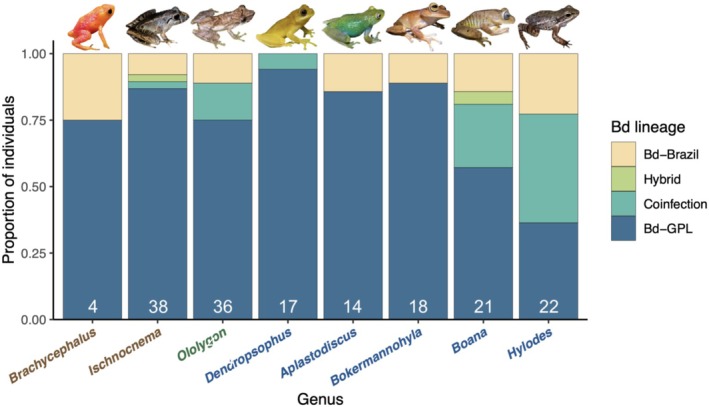
Stacked bar plot showing the proportion of samples assigned to each *Batrachochytrium dendrobatidis* (Bd) lineage from frogs found along aquatic transects only. Names of genera are colored by reproductive mode (brown: Terrestrial direct development, green: Larval development in vegetation, blue: Larval development in aquatic habitats). The number of samples for each genus is shown in white. Significant (*p* < 0.05) pairwise chi‐squared comparisons of Bd lineage counts: *Aplastodiscus* vs. *Hylodes* (*χ* ^2^ = 9.791, *p* = 0.007), *Boana* versus *Ischnocnema* (*χ* ^2^ = 8.254, *p* = 0.041), *Bokermannohyla* versus *Hylodes* (*χ* ^2^ = 12.679, *p* = 0.002), *Dendropsophus* versus *Hylodes* (*χ* ^2^ = 13.650, *p* = 0.001), *Ischnocnema* versus *Hylodes* (*χ* ^2^ = 20.322, *p* < 0.001), *Hylodes* versus *Ololygon* (*χ* ^2^ = 8.696, *p* = 0.013).

**FIGURE A3:**
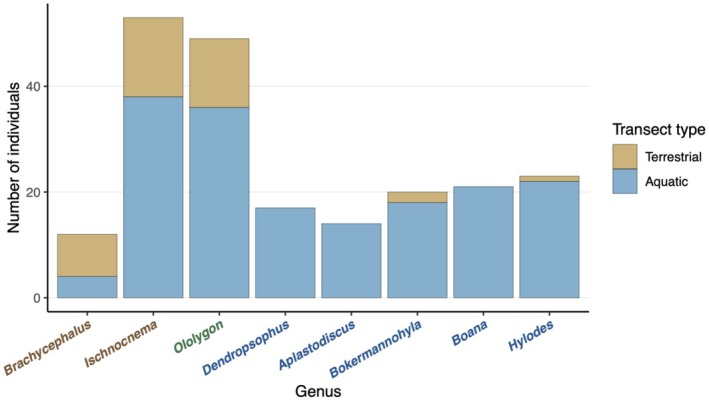
Stacked bar plot showing the number of frogs captured along each transect type from genera of frogs with more than *n* = 10 successfully genotyped swabs. Names of genera are colored by reproductive mode (brown: Terrestrial direct development, green: Larval development in vegetation, blue: Larval development in aquatic habitats).

**FIGURE A4:**
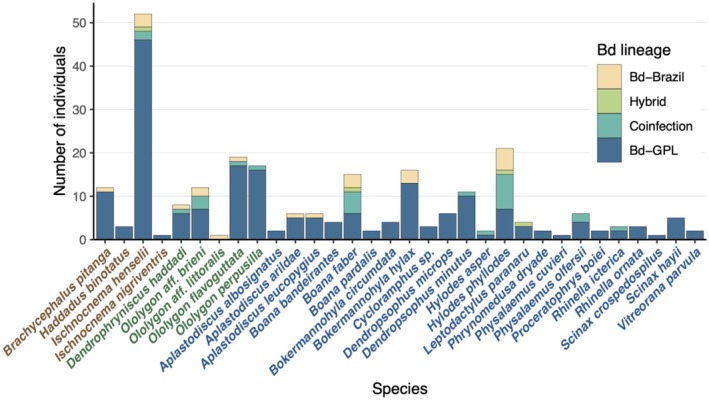
Stacked bar plot showing the number of samples from all frog species and their assigned *Batrachochytrium dendrobatidis* (Bd) genotype. Names of genera are colored by reproductive mode (brown: Terrestrial direct development, green: Larval development in vegetation, blue: Larval development in aquatic habitats).

We detected statistically significant pairwise differences in the distribution of Bd genotypes between certain genera (Figure 5). *Hylodes* had a higher likelihood of coinfection compared to *Aplastodiscus*, *Bokermannohyla*, *Brachycephalus*, *Dendropsophus*, *Ischnocnema*, and *Ololygon* (Figure 5). *Boana* also had a significantly higher likelihood of coinfection than *Ischnocnema* (Figure 5). These results held true when only considering frogs captured on aquatic transects, except for the significant difference between *Hylodes* and *Brachycephalus*, for which we did not achieve an adequate sample size (*n* = 4; Figure A2). The number of frogs from each genus caught along terrestrial and aquatic transects is shown in Figure A3. Bd lineage detections for all species are shown in Figure A4.

**FIGURE 6:**
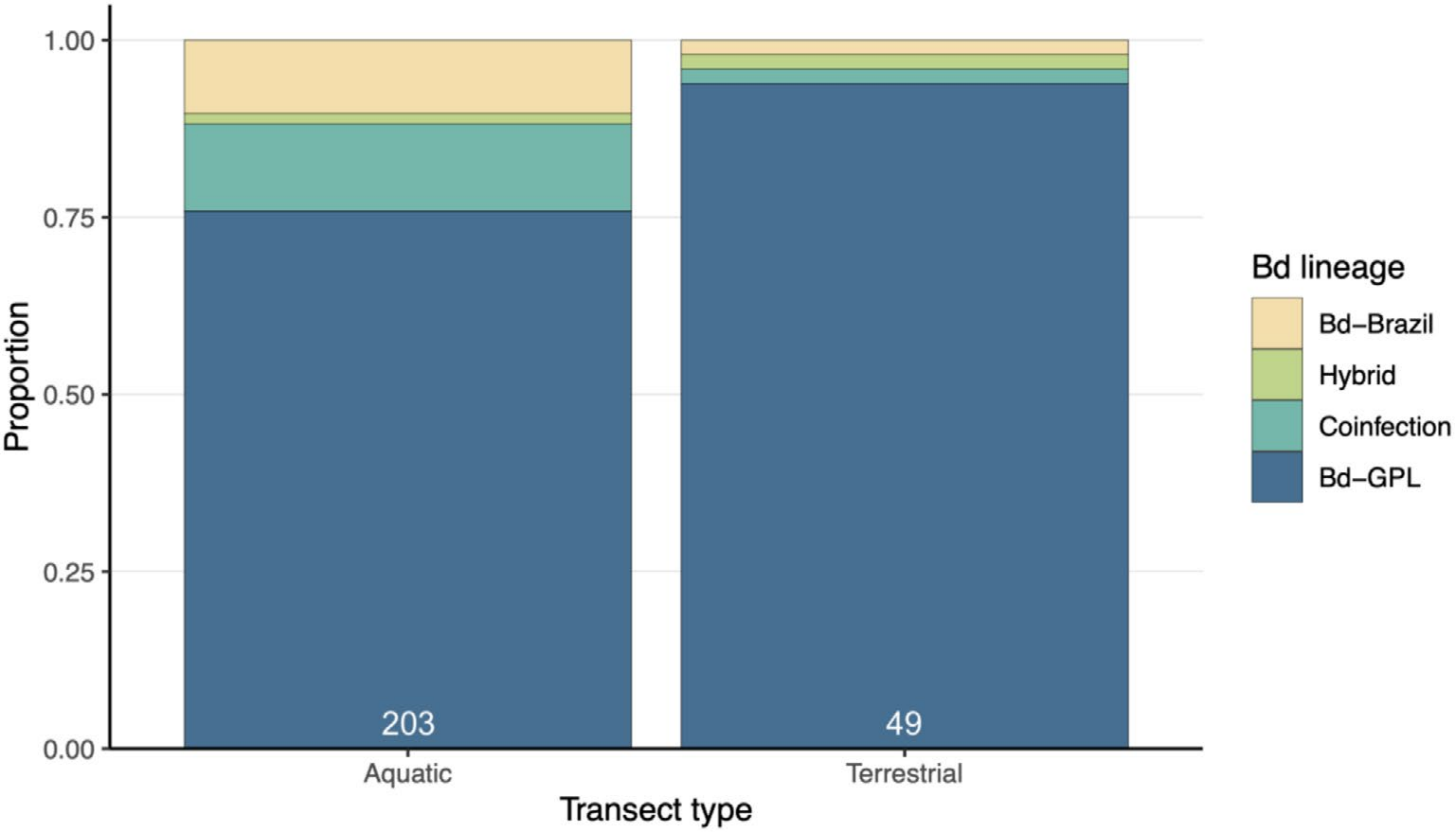
Stacked bar plot showing the proportion of each type of *Batrachochytrium dendrobatidis* (Bd) lineage detected on frogs captured along aquatic and terrestrial transects. The number of samples from frogs at each transect type is shown in white. Chi‐squared test of Bd lineage counts between transect types: *χ* ^2^ = 8.849, *p* = 0.031.

Amphibians captured along terrestrial transects almost exclusively harbored Bd‐GPL (Figure 6). Transect type was a significant predictor of whether Bd‐GPL was the only detected lineage, even when the amphibian species composition of the transect and host genus were included as random effects in the GLMM (Table A4). Developmental mode was not a significant predictor of the probability of single‐lineage Bd‐GPL infections (Table A4). No variables significantly predicted the presence of single‐lineage Bd‐Brazil infections (Table A5) or coinfections (Table A6).

## 4 Discussion

Our results demonstrate that *Batrachochytrium dendrobatidis* (Bd) lineage distribution is non‐randomly associated with both host genus and habitat type at our focal site in the Brazilian Atlantic Forest. We found significant differences in Bd lineage distribution primarily between the stream‐dwelling genus *Hylodes* and most other amphibian genera examined in this study. Specifically, *Hylodes* exhibited significantly higher rates of coinfection with Bd‐GPL and Bd‐Brazil compared to *Aplastodiscus*, *Bokermannohyla*, *Brachycephalus*, *Dendropsophus*, *Ischnocnema*, and *Ololygon*. We observed a striking pattern in Bd lineage distribution across terrestrial and aquatic transects, where amphibians captured along terrestrial transects nearly all had single‐lineage Bd‐GPL infections. Our models support this pattern, even when accounting for amphibian species composition and host genus, suggesting that aquatic habitats may harbor more numerous Bd lineages in this community.

Stream‐associated amphibians have experienced severe declines in Brazil and beyond, likely due to their occurrence in cool, moist habitats that favor Bd growth and prolonged contact with waterborne zoospores (Becker and Zamudio 2011; Carvalho et al. 2017). This pattern contextualizes our finding that *Hylodes*, a rheophilic genus with tadpoles that develop in stream water and adult males that defend stream territories (Heyer et al. 1990), exhibit the highest coinfection rates and thus serve as a reservoir for Bd lineage diversity. This lifelong association with streams promotes continuous exposure to zoospores from multiple lineages. However, our data cannot disentangle genus‐specific effects from habitat specialization in stream‐dwelling frogs like *Hylodes*, as we lack sufficient replication from other rheophilic genera as well as samples from other localities.

The tree frog genus *Boana* also exhibited a high incidence of coinfections. *Boana faber*, our most well‐represented species in this genus, is a large, pond‐breeding frog that is seasonally associated with water but migrates into the surrounding environment after the breeding season. However, during their time spent in more terrestrial habitats, *B. faber* frequently shelters in bromeliads (Neely et al. 2023). This behavior may lead to the spread of Bd genotypes by exposing bromeliad‐breeding frogs like *Ololygon* and *Dendrophryniscus* to lineages of Bd found in ponds and streams. Unmeasured differences in species' susceptibility to certain lineages of Bd may have also shaped the infection patterns we observed. Heterogeneity in the host immune response due to variation in MHC diversity (Savage and Zamudio 2011), antimicrobial peptide production (Rollins‐Smith 2023), and the skin microbiome (Muletz‐Wolz et al. 2017; Rebollar et al. 2020) might lead to disparate responses to certain Bd lineages between frog species.

Bd lineages are known to vary in thermal tolerance (Muletz‐Wolz et al. 2019; Sheets et al. 2021; Voyles et al. 2017), but less is known about differences in desiccation tolerance. The strong association between aquatic habitats and higher Bd lineage diversity in the present study suggests that aquatic environments may serve as refugia where Bd‐Brazil can persist alongside Bd‐GPL. This pattern raises the hypothesis that Bd‐GPL could be more desiccation‐tolerant, though alternative explanations include differences in host susceptibility and behavior between terrestrial versus aquatic frog assemblages, or historical variation in lineage invasion pathways. Experimental tests comparing differences in resistance to drying between Bd lineages may be key to understanding the findings described here and whether this is a site‐specific pattern or a universal property.

Based on our results, lineage competition may be occurring predominantly on aquatic frogs. Frequent contact with water might increase zoospore motility after release from sporangia, allowing them to re‐infect host skin farther from the original infection site (Berger et al. 2005). These conditions may also provide ample opportunities for recombination between lineages when infections bring multiple genotypes into close contact, allowing for parasexual or sexual reproduction, and the potential emergence of hyper‐virulent hybrid lineages (Nieuwenhuis and James 2016; Samarasinghe et al. 2020). Furthermore, recent evidence suggests that the divergence of many lineages predates documented amphibian declines (O'Hanlon et al. 2018), thus a combination of differences in pathogen traits along with global environmental factors (i.e., habitat modification and climate change) and complex invasion histories (i.e., multiple introductions) could be important, yet unexplored, factors in driving declines.

Several methodological limitations must be considered when interpreting our results. First, our detection of Bd lineages may be biased towards those that typically produce higher infection loads (Bd‐GPL; Jenkinson et al. 2018; McDonald et al. 2023), as our analysis revealed that genotyping success was strongly influenced by Bd load. Second, our genotyping approach was designed to be cost‐effective and allowed us to run nearly 800 samples at the expense of genetic resolution. Using two SNP assays does not provide sufficient resolution to differentiate between the full diversity of genotypes that may be present. It is important to note that this approach cannot reliably detect potential F2 hybrids, backcrosses, or hybrids from other Bd lineages, which would require more comprehensive genomic characterization like Bd‐capture or whole‐genome sequencing approaches (Byrne et al. 2017; Mulder et al. 2024; Rosenblum et al. 2013). The presence of such cryptic genetic diversity could influence the patterns we observed, particularly in hybrid zones like the one in São Paulo state (Carvalho et al. 2023). Additionally, we cannot rule out the possibility that environmental DNA from free‐living zoospores in water might contribute to detecting multiple lineages in aquatic frogs, though rinsing animals before swabbing them should reduce the probability of this source of error.

In conclusion, our study demonstrates that Bd lineage distribution in a hyper‐diverse tropical amphibian community could be shaped by a combination of both host taxonomy and habitat use, with important implications for understanding the persistence of less competitive Bd lineages and the emergence of hybrids in Brazil. Stream‐dwelling frogs, particularly *Hylodes*, appear to play a crucial role as reservoirs for the maintenance of both enzootic and invasive pathogen genotypes and may facilitate recombination between lineages. Our study provides valuable data on the host/habitat specificity and spatiotemporal dynamics of two important Bd lineages in South America. Future research would benefit from sampling at additional sites and employing higher‐resolution genotyping or sequencing approaches to uncover the full genomic diversity of Bd on individual amphibians. Such approaches may allow for more precise identification of multi‐generation hybrids and lineage‐specific adaptations that may influence host specificity and habitat associations. Understanding these multifaceted host‐pathogen dynamics is essential for predicting pathogen persistence and disease outcomes in diverse amphibian communities threatened by chytridiomycosis.

## Author Contributions

**Shannon Buttimer:** conceptualization (equal), data curation (equal), formal analysis (lead), investigation (equal), methodology (equal), visualization (lead), writing – original draft (lead), writing – review and editing (equal). **Wesley J. Neely:** formal analysis (equal), investigation (equal), writing – review and editing (equal). **Jack M. Boyette:** formal analysis (equal), investigation (equal), writing – review and editing (equal). **Carolina Lambertini:** investigation (equal), writing – review and editing (equal). **Renato Martins:** investigation (equal), writing – review and editing (equal). **Karen A. Paniagua Torres:** investigation (equal), writing – review and editing (equal). **David Rodriguez:** methodology (equal), resources (equal), supervision (equal), writing – review and editing (equal). **C. Guilherme Becker:** conceptualization (equal), funding acquisition (lead), methodology (equal), project administration (lead), resources (equal), supervision (lead), writing – original draft (supporting), writing – review and editing (equal).

## Funding

This work was supported by the National Science Foundation (BII‐2120084, DEB‐2227340, DEB‐2413542).

## Conflicts of Interest

The authors declare no conflicts of interest.

## Data Availability

The data and code that support the findings of this study are openly available on Figshare (https://doi.org/10.6084/m9.figshare.29695307).

## Appendix A

**TABLE A1:** Summary of the number of frog encounters per sampling campaign.

Campaign	Season	Date range	Sample size
1	Wet	December 2—December 15, 2020	224
2	Wet	February 10—February 20, 2021	203
3	Dry	June 17—June 20, 2021	86
4	Dry	July 27—August 6, 2021	198
5	Dry	September 13—September 27, 2021	579
6	Wet	October 11—December 4, 2021	586
7	Wet	February 11—February 22, 2022	332
8	Dry	May 3—May 13, 2022	316
9	Dry	July 10—July 29, 2022	305
10	Wet	October 12—November 26, 2022	543
11	Wet	*January 10—January 21, 2023*	464

**TABLE A2:** PERMANOVAs of Jaccard distances, comparing frog species composition by transect pair ID and transect type.

Distance metric	Explanatory variable	Pseudo‐F	*R* ^2^	*p*
Jaccard	Transect pair ID	6.947	0.202	**< 0.001**
	Transect type	22.077	0.128	**< 0.001**
	Transect pair ID × Transect type	4.694	0.136	**< 0.001**

*Note:* Bolded values are statistically significant (*p* < 0.05).

**TABLE A3:** *Batrachochytrium dendrobatidis* (Bd) prevalence and infection intensity for each sampled amphibian species from São Paulo, Brazil.

Amphibian species	Sample size	Number infected	Mean Bd prevalence (95% CI)	Mean Bd infection intensity (95% CI)
*Adenomera marmorata*	3	0	0.00 (0.00, 0.69)	—
*Aplastodiscus albosignatus*	8	4	0.50 (0.22, 0.78)	6.2 × 10^3^ (0, 1.7 × 10^4^)
*Aplastodiscus arildae*	34	13	0.38 (0.23, 0.56)	6.2 × 10^5^ (0, 1.9 × 10^6^)
*Aplastodiscus leucopygius*	33	12	0.36 (0.21, 0.55)	1.8 × 10^4^ (0, 3.7 × 10^4^)
*Boana bandeirantes*	82	25	0.30 (0.21, 0.42)	2.6 × 10^4^ (0, 6.0 × 10^4^)
*Boana faber*	49	27	0.55 (0.40, 0.69)	1.6 × 10^5^ (0, 3.5 × 10^5^)
*Boana pardalis*	3	2	0.67 (0.13, 0.98)	7.0 × 10^6^ (0, 9.4 × 10^7^)
*Bokermannohyla circumdata*	48	16	0.33 (0.21, 0.49)	2.3 × 10^4^ (0, 5.6 × 10^4^)
*Bokermannohyla hylax*	116	37	0.32 (0.24, 0.41)	2.1 × 10^5^ (0, 5.0 × 10^5^)
*Brachycephalus pitanga*	1512	170	0.11 (0.10, 0.13)	5.8 × 10^5^ (0, 1.5 × 10^6^)
*Cycloramphus* sp.	10	5	0.50 (0.24, 0.76)	8.1 × 10^3^ (0, 2.2 × 10^4^)
*Dendrophryniscus haddadi*	278	38	0.14 (0.10, 0.18)	7.6 × 10^4^ (4.7 × 10^3^, 1.5 × 10^5^)
*Dendropsophus elegans*	5	1	0.20 (0.01, 0.70)	9.2 × 10^2^
*Dendropsophus microps*	62	26	0.42 (0.30, 0.55)	2.8 × 10^4^ (0, 6.0 × 10^4^)
*Dendropsophus minutus*	70	31	0.44 (0.33, 0.57)	6.6 × 10^4^ (0, 1.4 × 10^5^)
*Dendropsophus seniculus*	1	0	0.00 (0.00, 0.95)	—
*Fritziana fissilis*	6	0	0.00 (0.00, 0.48)	—
*Fritziana ohausi*	5	3	0.60 (0.17, 0.93)	1.5 × 10^3^ (0, 7.9 × 10^3^)
*Haddadus binotatus*	25	4	0.16 (0.05, 0.37)	2.6 × 10^5^ (0, 5.4 × 10^5^)
*Hylodes asper*	9	6	0.67 (0.31, 0.91)	8.5 × 10^3^ (0, 2.6 × 10^4^)
*Hylodes phyllodes*	169	69	0.41 (0.33, 0.49)	6.8 × 10^4^ (0, 1.4 × 10^5^)
*Ischnocnema henselii*	232	95	0.41 (0.35, 0.48)	8.6 × 10^5^ (0, 1.8 × 10^6^)
*Ischnocnema nigriventris*	8	1	0.13 (0.01, 0.53)	3.3 × 10^6^
*Ischnocnema parva*	86	12	0.14 (0.08, 0.23)	3.3 × 10^2^ (0, 8.4 × 10^2^)
*Ischnocnema aff. lactea*	1	0	0.00 (0.00, 0.95)	—
*Leptodactylus paranaru*	9	7	0.78 (0.40, 0.96)	3.7 × 10^4^ (0, 1.1 × 10^5^)
*Ololygon aff. brieni*	90	34	0.38 (0.28, 0.49)	1.7 × 10^4^ (1.4 × 10^3^, 3.3 × 10^4^)
*Ololygon aff. littoralis*	8	2	0.25 (0.04, 0.64)	6.7 × 10^2^ (0, 6.6 × 10^3^)
*Ololygon flavoguttata*	69	35	0.51 (0.39, 0.63)	2.5 × 10^5^ (0, 5.3 × 10^5^)
*Ololygon perpusilla*	76	26	0.34 (0.24, 0.46)	1.1 × 10^5^ (0, 2.6 × 10^5^)
*Phrynomedusa dryade*	19	14	0.74 (0.49, 0.90)	4.7 × 10^4^ (0, 1.0 × 10^5^)
*Physalaemus cuvieri*	1	1	1.00 (0.05, 1.00)	2.1 × 10^3^
*Physalaemus olfersii*	29	8	0.28 (0.13, 0.47)	3.4 × 10^5^ (0, 8.7 × 10^5^)
*Proceratophrys belzebul*	2	0	0.00 (0.00, 0.80)	—
*Proceratophrys boiei*	92	17	0.18 (0.11, 0.28)	3.2 × 10^3^ (0, 7.1 × 10^3^)
*Rhinella icterica*	55	15	0.27 (0.17, 0.41)	3.6 × 10^3^ (0, 8.3 × 10^3^)
*Rhinella ornata*	9	3	0.33 (0.09, 0.69)	4.8 × 10^3^ (0, 1.2 × 10^4^)
*Scinax crospedospilus*	4	1	0.25 (0.01, 0.78)	2.8 × 10^6^
*Scinax fuscovarius*	1	1	1.00 (0.05, 1.00)	1.6 × 10^2^
*Scinax hayii*	45	15	0.33 (0.20, 0.49)	2.1 × 10^3^ (7.8 × 10^2^, 3.5 × 10^3^)
*Trachycephalus imitatrix*	1	0	0.00 (0.00, 0.95)	—
*Vitreorana parvula*	39	5	0.13 (0.05, 0.28)	5.5 × 10^2^ (0, 1.4 × 10^3^)
Overall	**3404**	**781**	**0.23** (**0.22, 0.24**)	**3.2 × 10** ^**5**^ (**8.5 × 10** ^**4**^, **5.5 × 10** ^**5**^)

*Note:* Bd infection intensity is given as ITS copies per swab. Confidence intervals (CIs) of mean Bd infection intensity are not shown for species with fewer than two positive swabs. Bolded values are statistically significant (*p* < 0.05).

**TABLE A4:** Results of a binomial generalized linear mixed model predicting the presence of a single‐lineage *Batrachochytrium dendrobatidis* (Bd)‐GPL infection.

Response variable	Explanatory variable	Estimate	*Z*	*p*
Bd‐GPL presence/absence	Species composition	−1.078	−0.937	0.3489
	Transect type (aquatic)	−1.716	−1.963	**0.0497**
	Developmental mode (terrestrial)	0.585	0.666	0.5051

*Note:* Fixed effects include species composition (Jaccard PCoA Axis 1) and transect type. Transect pair ID and host genus were included as random effects. Bolded values are statistically significant (*p* < 0.05).

**TABLE A5:** Results of a binomial generalized linear mixed model predicting the presence of a single‐lineage *Batrachochytrium dendrobatidis* (Bd)‐Brazil infection.

Response variable	Explanatory variable	Estimate	*Z*	*p*
Bd‐Brazil presence/absence	Species composition	2.187	1.823	0.0682
	Transect type (aquatic)	22.926	0.001	0.9994
	Developmental mode (terrestrial)	−0.307	−0.477	0.6333

*Note:* Fixed effects include species composition (Jaccard PCoA Axis 1) and transect type. Transect pair ID and host genus were included as random effects.

**TABLE A6:** Results of a binomial generalized linear mixed model predicting the presence of coinfection with *Batrachochytrium dendrobatidis* (Bd)‐Brazil and Bd‐GPL.

Response variable	Explanatory variable	Estimate	*Z*	*p*
Coinfection presence/absence	Species composition	−0.917	−0.538	0.5908
	Transect type (aquatic)	0.475	0.395	0.6925
	Developmental mode (terrestrial)	−1.621	−1.040	0.2984

*Note:* Fixed effects include species composition (Jaccard PCoA Axis 1) and transect type. Transect pair ID and host genus were included as random effects.
